# Using Low-Cost Sensors to Develop a High Precision Lifting Controller Device for an Overhead Crane—Insights and Hypotheses from Prototyping a Heavy Industrial Internet Project

**DOI:** 10.3390/s18103328

**Published:** 2018-10-04

**Authors:** Heikki Sjöman, Juuso Autiosalo, Jari Juhanko, Petri Kuosmanen, Martin Steinert

**Affiliations:** 1 Faculty of Engineering Science and Technology, Norwegian University of Science and Technology, Richard Birkelandsvei 2B, 7491 Trondheim, Norway; martin.steinert@ntnu.no; 2 Department of Mechanical Engineering, Aalto University, Otakaari 4 (K1), 02150 Espoo, Finland; juuso.autiosalo@aalto.fi (J.A.); jari.juhanko@aalto.fi (J.J.); petri.kuosmanen@aalto.fi (P.K.)

**Keywords:** Internet of Things, Industrial Internet, prototyping, Wayfaring, iterating

## Abstract

The subject of this study was the product development project creating a new innovative proof-of-concept (POC) prototype device that could control a connected industrial overhead crane in order to perform automatic or semi-automatic high precision lifts within a limited time frame. The development work focused on innovating a new measuring concept, which was parallel to finding suitable sensors for the application. Furthermore, the project resulted in a closed loop control system with Industrial Internet connected sensors and a user interface for factory workers. The prototyping journey is depicted to illustrate the decisions made during the product development project to contribute to both the pragmatic and the process discussion in the field of Industrial Internet. The purpose of this research was to explore and generate hypotheses for how new applications should be developed for heavy industry connected devices. The research question is: what are the implications of applying agile product development methods, such as Wayfaring, to heavy industrial machinery and Industrial Internet -based problems? The methodologies used in this paper, in addition to developing the device, are case study research and hypotheses generated from case studies. The hypotheses generated include that it is also possible to prototype large size connected machinery with low-cost and in a short time, and investment decisions for heavy Industrial Internet products become easier with concrete data from proof-of-concept prototypes by creating knowledge about the investment risk and the value proposition.

## 1. Introduction

**Figure 1:**
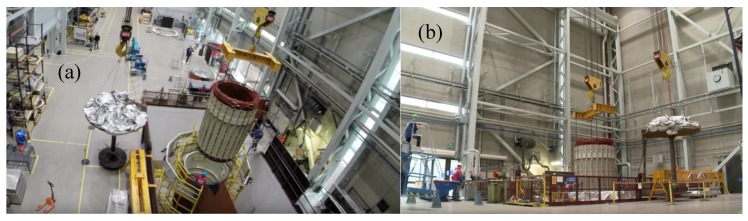
The original shrinking fit lift operation to be automated in the project. Screen captures are taken from a video of the assembly [2]. The capture (**a**) is the view of the operation from above at the time 0:24 and the capture (**b**) is a side view at the time 0:26 of the video.

In the industrial context, the usage of fully automated cranes is still quite rare because of the unclear relationship of predicted investment costs and the perceived cost savings [1]. The managers of the case company faced the problem of performing a particular manually controlled heavy shrink fit assembly lift operation. A large (over 100 ton) electric motor must be reliably fitted (with high precision of under 0.5 mm) within a limited time frame (five minutes) before the heated part cools down and closes the gap. The initial reception for this case was disbelief that anything could be achievably built that would not cost a lot and take a lot of time while still being uncertain about the advantages. The common opinion was that crane applications lifting 30–100-ton objects must be too large and heavy to prototype. The subject of the automated lift is depicted in Figure 1 from above and from the side.

The idea behind this study was to create a proof of concept solution to simplify and solve this lifting problem. During the course of this project, we realized that we had developed insights that could possibly be utilized in other similar projects, so we began generating hypotheses for the very early stage: the ‘fuzzy front end’ of product development of applications for heavy industry connected devices [3]. The linkage between the flexible design process and firm competitiveness in the early phase was discussed extensively in the software engineering process community whilst adopting agile methodologies [4], as shown in [5,6]. Industrial Internet, as a complex field of mixing digital and physical development, would benefit from similar discussion [6]. By doing the case by ourselves, the detail of research data became very rich. We created the hypotheses based on building the prototypes, showing the thinking behind the whole development process and finally our reflections on it. The case is in hypothesis format so that other researchers and practitioners can then further explore, test, and build upon it.

The reason that the lift was so difficult to perform with an overhead crane is that the problem is physically very complex and is influenced by several dynamic factors. The wires carrying the load are dynamically stressed and worn based on the movement while the load, temperature, current length of the wires, elasticity of the wires, wear factor, and time from the last maintenance all contribute to the location of the hoist and therefore the location of the load [7]. These factors are hard to measure and thus are also hard to mathematically model and predict. Also, minimizing the operation time and maximizing precision are partially conflicting requirements since the swing of the payload is dependent on the acceleration of the trolley [1].

Whilst the modelling is hard, it is also hard to achieve very good precision of the load at the very end of the chain of the crane parts (bridge, trolley, wires, and hoist) by only measuring the location of the individual parts. The general precision of locating loads with this particular crane is, according to the manufacturer, within one to two centimeters. The Industrial Internet connectivity and the digital twin of the crane helps the user or the developer to access the known information of the dynamic conditions of the crane and, through the application programmable interfaces (APIs), to build custom control systems in order to control the crane in a more fine-grained way.

We define Industrial Internet (II) as Internet of Things (IoT) used in a professional setting in industry. The definition of IoT has changed over the years of its existence [8], but generally with IoT we mean a product with sensors that measure some of the properties of its environment that sends this data via wired or wireless link to a receiver that may use it in conjunction with data from other products to change the environment via actuators. Additionally, when connected equipment or their loads are weighing tons instead of kilos we refer to the heavy Industrial Internet. Using this data, either a human or another device can act upon said insights, in our case when locating the load of the crane. What is not clear is whether there is a difference between how IoT/II products should be approached and what the implications and characteristics of Industrial Internet are in the realm of new product development. In such products there are two sides: the requirements for the product itself and the requirements for the insights it produces. These two are in constant interplay with each other when the product is in concept phase. The prototyped product yields a set of values that can be interpreted, combined, and processed in many ways into insights. The insights should be prototyped as well and tested with the users [9]. Prototyping the insights with low-cost sensors creates more ideas that could change the definition and the use of the product as well as being an effective way to confirm and reject core technologies to be used within the project.

In this paper, we discuss how we reviewed, compared, and built multiple prototype iteration rounds with low-cost sensors in order to solve the aforementioned engineering design problem and to help the managers to make more informed investment decisions for research and development based on the case. The results contain hypotheses that, after testing, are intended to contribute to the theory of how prototypes are used within the context of new product development in the heavy Industrial Internet.

## 2. Materials and Methods

Studying correct and sensible hypotheses is an important step when developing theories in their early stages, as has originally been pointed out by Edmondson and McManus [10] and later backed by Lager [11]. We follow the methodology of Yin for studying cases [12] and consequently Eisenhardt for building theory from case studies [13]. Otherwise, we are following the general guidelines of engineering design research of Blessing and Chakrabarti [14]. The research contributes to characteristics of how new product development, with methods like Wayfaring [15,16], could work in early stage projects by hypothesis generation and evaluation in the context of heavy industrial machinery and Industrial Internet/Internet of Things. We aim to follow the elements of the existing methodology of mindful and structured iterative design/build/test prototyping for the product development and understand how they are suited to this particular context. The methodology has formalized some of the findings of Lopez et al. for low cost sensor platform development, such as using theoretical approaches, simulations, and prototype development as intermediate solutions to reach the final sensor system [16]. In Wayfaring methodology these prototyping rounds, called ‘probes’, guide the development process further after each testing and form a journey in hindsight. The prototyping journey of this project is described in Section 4. The probes in the mechanics discipline followed a structure that inherently included the three steps of designing, building, and testing as defined by Gerstenberg et al. [17]. Similarly, many probes within the user interface, software, and electronics disciplines produced tangible results that required all of these three steps, as in the original case study of Wayfaring [18]. However, the project also included different kinds of work aggregates—such as brainstorming, reviews, and a site visit—which are added as independent probes as well. The complementary probes are essential for understanding the progress of the project, and are therefore added among the probes following the original idea more clearly. Some ideas need no concrete prototypes to be analyzed, but they can nevertheless spur new directions for the wayfaring journey. Therefore, not all probes aimed to produce a tangible result, but provided information in other ways, such as reviewing possible methods for certain issues. The hypotheses are generated and proposed as the results based on insights and reflections of the product development case presented in this paper.

In the product development in-depth case study, the authors followed the following systematic methodology: writing down the plan for the week each working week and reflecting and writing notes at the end of every week - or even a day - about how the prototyping process went. The authors documented the progress of the project using multiple methods, including writing notes, taking pictures and videos, writing short-term action plans, and using version control software for the programming activities. Furthermore, as the project took rather a long time when including pauses between effective working time, time stamps from communication tools and file systems were used in hindsight to track the exact timing of actions if the documentation was not sufficient. The documented progress was then analyzed to divide the work into aggregates fitting in the description of a probe in the Wayfaring method. Although many of the probes were implemented purposefully to follow the Wayfaring method, some of them were carried out due to necessity or intuition.

There are several advantages of the researchers acting as the workers at the same time, most importantly the intensity of details, problems and solutions that are captured during researching the cases. The method is used effectively when the researchers have access to a research community at the same time as they are working on an industry case, because the down side of the method could be no research at all if adequate reflection is not harnessed. The focus of the method is to bring scientific findings to practitioners in the industry.

At the end of using the Wayfaring methodology, there is a handover to a team with a more traditional engineering approach, such as the stage gate model [19] or waterfall model [20], that implements and engineers the product to the final form based on the requirements defined at the end of the fuzzy front end.

## 3. The Case

**Figure 2:**
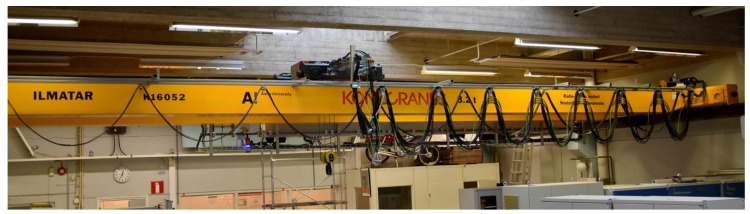
The Industrial Internet overhead crane used in the research.

The starting point of the project was an Industrial Internet capable crane donated for the purpose of generating new knowledge around the crane as a platform, depicted in Figure 2 and described in [21]. The academic team started by gathering interesting opportunities from industry partners. A suggestion for a problem was received from an assembly company to create a controller device for their shrink fit procedure to assist or perform the lift required. The problem was evaluated by visiting the case company and interviewing the workers and the management. Also, the manually controlled lift operation was video recorded in order to understand the problem better and be able to relive and describe the lift for all the stakeholders and the broader audience. In this case, the company wanted help in one of their lifting processes to create an either fully or semi-automatic controller device for workers of the factory in order to achieve better reliability, transferability, and precision of the operations at the assembly. This particular lift is an assembly operation where a large electric motor is lifted inside a motor frame. Typically, an expert operator uses an overhead crane to manually perform the lift while four other skilled workers are acting as the eyes and hands of the operator and helping to fulfill the precise requirements of the lift. The frame is heated for the shrink fit assembly and thus momentarily provides only a maximum 0.5 mm of play between the stator and the frame. This creates interesting requirements for the lift in that it needs to be so precise under a short time limit of five minutes. The discussion of the goal of the project circled around the creation of a ‘parking aid’ for the crane with which the operator could see how close the load is from the target. Another practical requirement was that the setup time of the ‘aid’ should be minimized. In the end, the aim for the project became to create a piece of equipment that would perform the lift as automatically as possible.

The resources for this project were a total of seven weeks of design, development and building time from a two-person group and material costs of around 200 euros (additionally the cost of an external experimental setup, that will serve many projects). The project resulted in a development platform for high accuracy positioning with an overhead crane and the first proof-of-concept solution for it. The exact composition of the technical realization is not the subject of this paper and is described here only on a project level.

### 3.1. Experimental Setup

**Figure 3:**
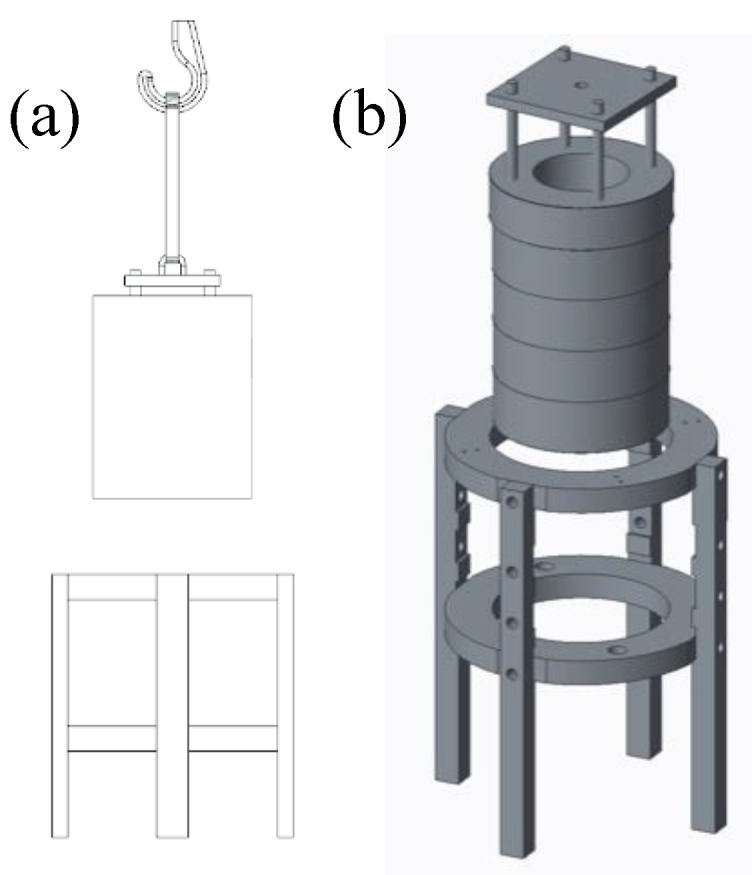
The physical experimental setup for testing the precision of a sensor concept. There is a 2D blueprint (**a**) on the left and rendered 3D model (**b**) of the setup on the right.

In order to measure the performance of any future proof-of-concept prototypes, we needed to build our own test rig. The experimental setup consisted of a crane with a 3.2 ton lifting capacity and a 60 kg special machined cylinder with four different tight fits in relation to a pile of rings that provide required fittings suitable for the task at hand. The rings were machined and attached to each other with four poles and bolts, as in Figure 3. The precision requirements were given by the case company and transformed to the scale since the tolerances are proportional to the scale. We also added one tighter extra step of an ‘impossible’ precision requirement to test the limits of any given control system explored. During the project, the testing was performed continuously by seeing whether the current iteration of the controller device could satisfy the requirement of lifting the cylinder inside the rings.

### 3.2. Enablers of the Project

To enable exploring the different opportunities, the proper access control for controlling the crane was created and given to the researchers over the developed OPC Unified Architecture (OPC UA, [22]) API. OPC UA is a protocol that is used to control the crane on a very low level, such as by giving the percentage of max voltage to the motor drives. In order to drive the crane with a modern high-level programming language, the python API was created on top of the OPC UA layer by the authors. This enabled the authors to develop applications for the crane much faster.

From the health and safety point of view, since the crane is potentially harmful equipment when it is driven programmatically, a sandbox environment was created where the top speed of the crane was set to 40% of the real maximum speed. The crane also had physical limit switches that prevented it from crashing into a wall. The normal radio controller of the crane was altered to act as a dead man’s switch (hold-to-run) and thus two buttons needed to be pressed down so that any input code could control the crane. This ensured the safe operation of the crane while being flexible to the needs of a control system.

## 4. Description of the Wayfaring Journey

The post-project analysis led to the identification of probes listed in Table 1. The probes are categorized into five streams according to their disciplines: (1) discipline-independent, (2) user interface, (3) software, (4) electronics, and (5) mechanics. Some of the probes had long-term actions or had results that were used for a long time, which is why some probes are shown multiple times. The probes were evaluated after the project by determining if the result of the probe was used as part of the final prototype (v) or discontinued (x). Probes that provided results that other probes continued were classified as neutral (-).

**Figure 4:**
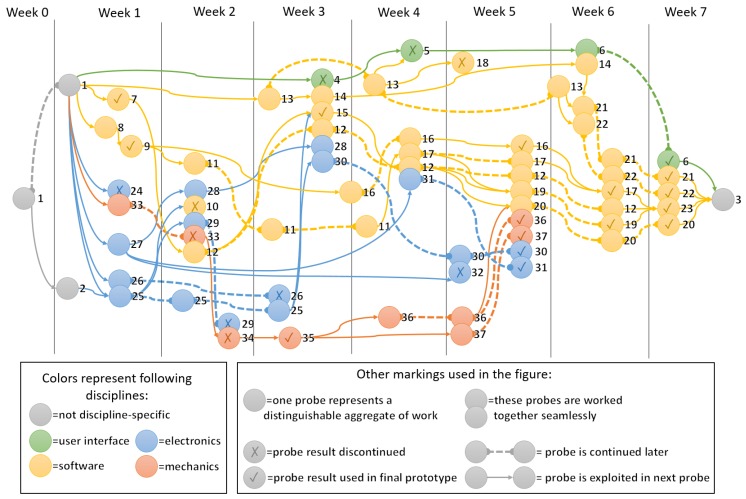
Wayfaring journey depicted with probes.

The wayfaring journey of the project is depicted in Figure 4 with probe numbers explained separately in Table 1 and each probe are explained in more detail later in this section. As the probes were identified after the project, so the figure was created afterwards. Some leads that were not properly documented may not have been visualized here although they have probably affected the course of the project, as decisions of next steps are made under uncertainty, partially based on intuition, in the Wayfaring methodology. Nevertheless, the Figure 4 based on probes does portray the steps and decision chains of the project at a glance more accurately than many other data visualization techniques would.

### 4.1. Progress of the Project

The work was implemented in one-week intensive periods with several weeks between each working week. Not all the working weeks are exactly one week in length, but all of them are equal to the approximate work contribution of one week, with the exception of the preparatory Week 0. The weeks are described in Section 4.1.1, Section 4.1.2, Section 4.1.3, Section 4.1.4, Section 4.1.5, Section 4.1.6, Section 4.1.7 and Section 4.1.8 and a reader can refer to Table 1 and Figure 4 while reading the descriptions. The table features a description of the core task and numbering of each probe and the figure shows relations to other probes.

Week descriptions included the following points:

- Overall aim and result of the week.
- Considerations on working structure of the week: e.g., was the work implemented in consecutive days.
- Probe descriptions as a bullet point list.
- Combinations of the probes.

#### 4.1.1. Week 0

The project started in Week 0 by searching for a use case to apply the Wayfaring methodology. Industrial Internet focused research work began around the overhead crane that was about to be donated for the university. As result, the use case was found from Finnish heavy industry and the project workers were identified.

The week was not formulated as a proper working week of the project with a clear start and end, but it rather included the preliminary ideation and conversations from quite a long time before actual development work. This kind of preparation is required before any kind of project can be started. In this project, Week 0 was initiated by the authors.

Week 0 included the following probe:

• Probe 1 included initial benchmarking and brainstorming of the upcoming project as well as negotiating the official start for the project. A high accuracy assembly procedure from Finnish heavy machinery industry was identified as the use case of the project. To further specify the use case, Probe 1 was continued on Week 1.

#### 4.1.2. Week 1

Week 1 started the actual development work of the project with the target of clarifying the previously identified use case and probing the first possible solutions for the identified problem. As a result, the need was documented, reviews and tests were conducted, and even some software related decisions that lasted to the final POC were made.

This included different types of working such as concrete workshops and conducting technology surveys, although general project benchmarking continued. Week 1 was also fragmented, meaning that the work was not on consecutive working days, but was divided into several actual working weeks. This was mainly due to the nature of the work, which included company visits that naturally must be organized when it is suitable for the employees of the case companies. Nevertheless, Week 1 is a clearly distinguishable aggregate of work. (16 November–2 December).

In order to create the control feedback loop on the exact position of the lifted object, the position of the object had to be measured accurately. Hence, a brief survey of possible sensors was conducted. The survey provided the following five types of sensors, categorized by their operating principle: Laser sensor, infrared sensor, ultrasound sensor, camera with feature detection, ultra-wideband (UWB) radar, and induction (Hall effect) sensor. More exact considerations are given in Table 2. Following the quick probing cycle methodology of the Wayfaring, infrared and ultrasound sensors were left out at very early phase because of not providing high enough accuracy at the low cost. The reasoning for not to choose laser sensors were difficulties while used with bright surfaces and high price. UWB radar as a measuring technology turned out to have very noisy signal especially with metallic surfaces. A thermal camera was too expensive to prototype and shared the same difficulties with a normal camera. Later, the induction sensor was left out because of the high price, following the target of low investment costs for the first phase of the project. However, the induction sensors were left for consideration as the potential sensor choice for the later phases. Hence, a laser sensor and camera detection were selected for further development. Both of these categories include expensive high-end products, but also low-cost alternatives suitable for testing an overall concept and user interaction. The development started with acquiring cheap sensors (a time of flight sensor (TOF) and a webcam) which were compared in parallel by creating incrementally enhanced testing of the sensors, starting from simple tests on an office table and leading to bigger scale tests. During the tests, the camera proved to be impractical for measuring high accuracy in this application. The TOF sensor was therefore selected for further prototyping.

Week 1 included following Probes 1, 2, 7, 8, 9, 24, 25, 26, 27, and 33:

- Probe 1 was started on Week 0 and was finished on Week 1 as the basic resources and main goal of the project were identified. The results (such as concrete ideas and topics for further investigation) of Probe 1 were distributed to the next probes.
- Probe 2 included two visits to the assembly company and taking videos of the assembly procedure. Furthermore, informal interviews were conducted to understand the problem from a factory worker (user) perspective. The results of this probe were used throughout the rest of the project to steer the development in an interpreted direction.
- The purpose of Probe 7 was to select the main programming language of the project. The review was relatively short and based mainly on the previous experience and knowledge of the workers. As result of the review, Python was selected as the main language thanks to its versatility, active development community, relatively high level of abstraction, and general ease of use. Python was used throughout the rest of the project, although the version was changed. Java language was another strong candidate.
- Probe 8 reviewed the alternatives for controlling the crane externally. The results of the review included (1) opening up an application programming interface (API), (2) modifying the software of the controller, and (3) modifying the hardware of the crane controller.
- Probe 9 represents the presentation of Probe 8 results to the crane company representatives, which led to the selection of OPC UA as the control interface for the crane. The basic specifications of the interface were agreed and the crane manufacturer later provided the feature description for controlling the crane through an open API.
- Probe 24 was a short test, inspired by work of Porazzo et al. [23], for evaluating whether the use of light could help a sensor to detect the width of the gap between the stator and the frame. The idea of the probe was not continued.
- Probe 25 reviewed the potential sensor types that could be used for detecting the gap between stator and frame. This is one of the most essential probes for achieving a successful project result. The reviewing work of the probe continued all the way to Week 3.
- Probe 26 included brainstorming and reviewing the possible methods for the automatic calibration of sensors that are attached to the frame to measure the gap between stator and frame. The probe provided three alternatives for the automatic calibration procedure: (1) long distance laser, (2) mechanically moving the sensor to measure the frame, and (3) accurate mounting holes for the sensors. The results were used in Probe 25 as the requirements for the sensors.
- Probe 27 implemented a short review of alternatives for the computation hardware of the prototype. Results, Raspberry Pi and Arduino, were used in Probes 31 and 32 consequently, although a laptop computer was also used in Probe 28 during development.
- Probe 33 started the mechanics stream by introducing the first physical miniature models of stator–frame combination and simultaneously reviewed the options for the miniature prototype that could prove that the basic idea of the project is viable. The first physical models included common objects found in an office, such as bottles, cans, and sheets of rubber. Options for the final test rig included a smaller stator–frame combination from the case company or self-designed and machined prototype with similar features. The experience of the probe was utilized in Probes 34 and 36.

The week had two small combinations of probes. Probes 24 and 33 were combined to see if light could be used as visual aid for sensors, and Probes 25 and 26 provided a combined review of sensor selection and self-calibration method.

#### 4.1.3. Week 2 (Probes 10, 11, 12, 25, 28, 29, 33, and 34)

The aim of Week 2 was to review OPC UA tools, build some test pieces, make a simple prototype, and to try out image recognition. All of the objectives were worked during the week, although none of them arrived at a final answer. Furthermore, Week 2 provided a clear start for the software development of the project. Week 2 was executed during one full calendar week (16–20 January).

**Figure 5:**
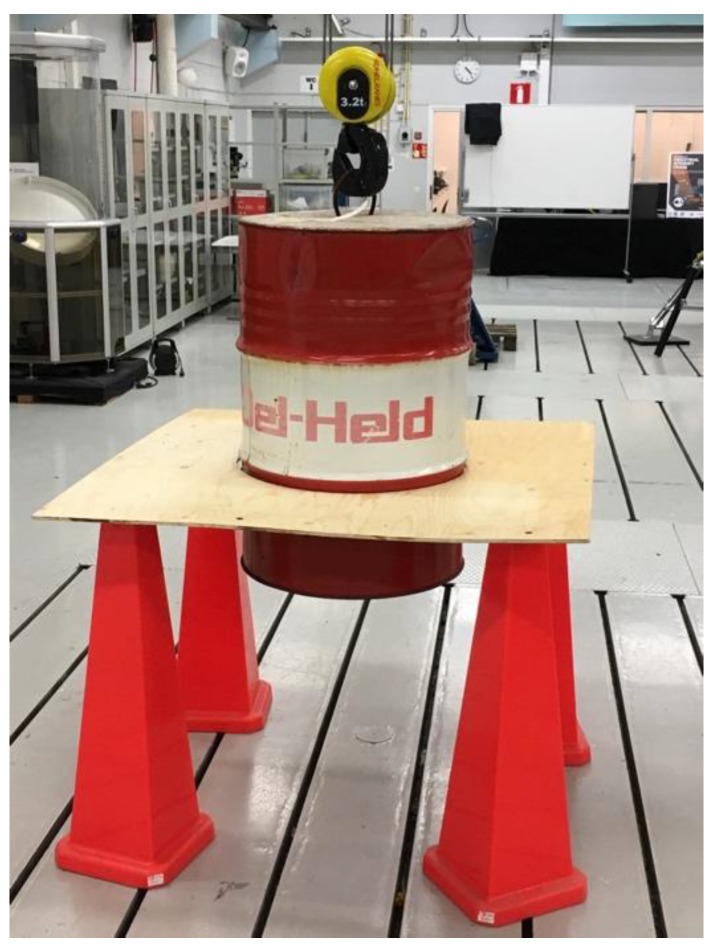
Early prototyping and concept creation for the test rig. Probe 34.

Week 2 included the following probes:

- Probe 12 introduced Python programming language to the project as it was used for using OpenCV library.
- Probe 11 started the review of possible OPC UA tools for future use.
- Probe 10 represents the use of OpenCV image processing library for detecting the location of round shapes from a camera feed with an intention to use it as control signal.
- Probe 29 provides a camera as a sensor to deliver images for OpenCV.
- Probe 33 continued the mechanics stream by using cylindrical metal cans as low-resolution mechanical prototypes.
- Probe 34 introduced a large-scale test rig that was constructed from oil barrel and plywood, seen in Figure 5. The accuracy of the test rig was seen as too low and the probe was discontinued.
- Probe 25 continued the review of possible sensors, and as result, ToF and webcam were selected for first round testing.
- Probe 28 provided laptops as the computing platform during the week.

The image detection prototype combined five probes (28, 10, 29, 33, 12) seamlessly together as the working prototype required laptop, OpenCV, camera, can, and Python simultaneously. Also, the camera (Probe 29) and oil barrel test rig (Probe 34) were tested together.

#### 4.1.4. Week 3 (Probes 4, 13, 14, 15, 25, 30, and 35)

The main goals and contributions of Week 3 were to prototype the integration of the data flow of a sensor to the user interface, and to design a high precision test rig. Also, minor technical issues were solved. The week was carried out during one full calendar week (6–10 February).

Week 3 included the following probes:

- Probe 4 built the first simple graphical user interface in order to understand how the data could be presented for the user and potentially used as an automatic control signal.
- Probe 13 reviewed software technologies to enable the UI visualization. The alternatives included HTML, Python flask, Curses, Tornado, Express, ExpressIO, JavaScript, socket, and WebSocket. The results were tested by the authors in Probes 4, 5, and 14.
- Probe 15 integrated measurement readings of a ToF sensor to Python code via open source libraries.
- Probe 25 concluded with a review of sensor alternatives presented in Table 2. The aim of the review was not to find the absolute best possible sensor, but to provide a good enough sensor for building a proof-of-concept prototype. The selection criteria emphasized availability, price, and usability rather than resolution, accuracy, or precision. After this project, one result of the review, eddy-current sensors, was tested in a follow-up student project. The results with the most potential (time of flight laser sensor and the camera) were tested in Probes 30 and 29 respectively.
- Probe 30 provided the ToF sensor and the electrical connections to Raspberry.
- Probe 35 gave an important step to the mechanics stream by designing a test rig that would serve as a prototyping platform and as a tool for measuring positioning performance. It consisted of two rings and a cylindrical test piece lathed with great precision to tolerances that would allow the authors to test whether the control system would work as anticipated.

Probes were developed together as the week featured a simple prototype with the complete information flow from sensors to the user interface. This chain required the simultaneous contribution of Probes 4, 14, 15, 12, 28, and 30.

#### 4.1.5. Week 4 (Probes 5, 17, 31, and 36)

The main aim of the week was to control the crane with the newly received OPC UA API and secondary aims were to control the crane based on image recognition, to test preliminary laser sensors, and to acquire new laser sensors. The main aim was reached and secondary aims were not.

The work on Week 4 was carried out during one full calendar week (27 February–3 March).

Week 4 included the following probes:

- Probe 16 introduced the OPC UA interface of the crane to the project. The crane manufacturer provided the interface just before Week 4 and the structure of the interface was studied and necessary software tools were installed.
- Probe 5 was an UI created with Curses library to serve the need for two-way communication between the prototype and the user.
- Probe 17 introduced an open source library, FreeOpcUa [24], to allow access directly from Python to the OPC UA interface. This enabled access to information and low-level control logic of the crane with Python code, and a need for higher-level abstraction was recognized.
- Probe 31 provided Raspberry Pi as a computing platform for the Python code.
- Probe 36 was started as the first part of the machined test rig was finished.

The week featured controlling the crane from external API, which required the contribution of Probes 5, 16, 17, 12, and 31.

#### 4.1.6. Week 5 (Probes 18, 19, 20, 31, 32, and 37)

The main task of Week 5 was to develop the first proof-of-concept application with subtasks to enable control, measurement, visualization, and integration of the previous subtasks. The control and measurement goals were reached, visualization only partially, and integration was not implemented. Week 5 was executed during one calendar week (21–24 March).

**Figure 6:**
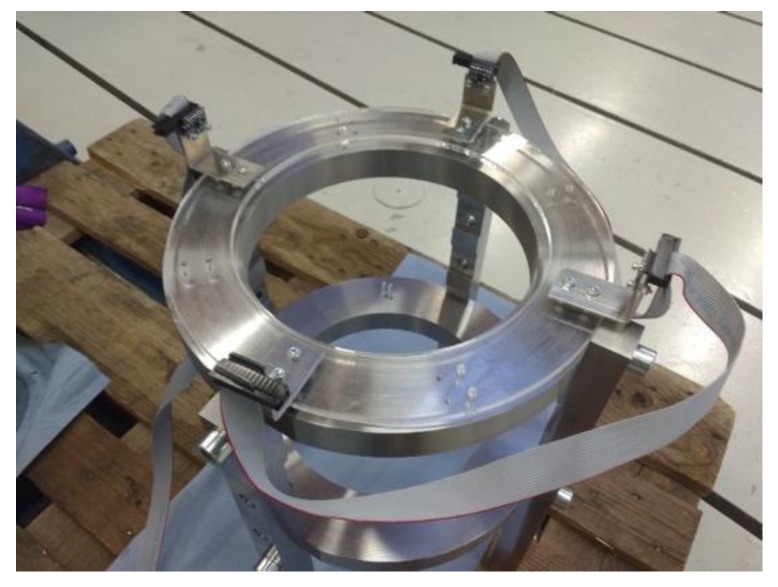
The top view of the Probe 37, which was also the final physical form of the prototype.

Week 5 included the following probes:

- Probe 18 tested a JavaScript library that would have allowed any mobile phone to act as a joystick. However, the authors decided to pursue an automatic solution and the joystick development was not continued.
- Probe 19 created our own class for the crane in Python so that we could control the crane on a higher abstraction level. The class was necessary because controlling the crane was a painful job programmatically as the API offered only attributes to control the direction and percentage of full power fed to the motors of the crane and an opportunity to read its internal coordination system. Safety features also created extra trouble, as the watchdog value had to be constantly updated (if the connection was lost, the non-updated watchdog value would stop the crane). The new class contained functions to move the crane to a certain place in the coordination system as well as connect to the crane and update the safety watchdog, not to mention the custom algorithms to move the crane very precisely.
- Probe 20 started the development of accurate control of the crane based on ToF sensor readings.
- Probes 30 and 32 tested the ToF sensors and Arduino together.
- Probe 37 fabricated a platform for four ToF sensors to be attached to the test rig frame, depicted in the Figure 6. The sensor platform was laser cut with appropriate fastening holes so that the sensors could have a fixed reference point in order to measure the distance between the simulated stator and the frame.

The main task required parallel work of many probes to create the proof-of-concept prototype. Therefore Probes 16, 17, 12, 19, 20, 36, 37, 30, and 31 were implemented together although full integration was not reached. Furthermore, the week had two minor actions with the combinations of Probes 30 and 32 to test ToF sensors with Arduino and Probes 36 and 37 to assemble the full mechanical test rig.

#### 4.1.7. Week 6 (Probes 6, 21, and 22)

The week focused on control and UI development with the goal of creating a working proof-of-concept demo. Previous probes were developed further and integrated to form one prototype, but the performance was not sufficient to provide a final proof-of-concept prototype. Week 6 was divided in to five days over three calendar weeks, but the total working hours equaled approximately one week (27 April–9 May).

Week 6 included the following probes:

- Probe 6 pursued the idea of dividing the lift into phases (approaching, lowering down, high precision location, final lowering). A simple web interface allowed it to move back and forth between these tasks.
- Probe 21 introduced a Tornado webserver that provided information from sensors to the user and relayed user commands back to the control logic. A switch-like part of the web server was created to send messages to the right addresses without flooding all the clients (mobile phone or web browser of the computer and crane control logic code running on Raspberry Pi) that would have otherwise lowered the performance of the server.
- Probe 22 provided websockets as a technical implementation to convey information between Python crane control logic code, Tornado webserver, and the user‘s web browser.
- The code started to become more and more complex so a versioning system git was started through GitLab service.

The main goal required integrated development of multiple software Probes, 21, 22, 17, 12, 19, and 20. Also UI development was carried out as the combination of Probes 6 and 14, and UI enabling technology was developed by combining Probes 21 and 22.

#### 4.1.8. Week 7 (Probes 3, 6, 20, and 23)

The week aimed to present the final proof-of-concept for company representatives. This required work on the user interface and control software. The goal was met and the project ended with the working proof-of-concept prototype. Week 7 was executed during one full calendar week (31 July–4 August).

Week 7 included the following probes:

- Probe 6 finalized the user interface that took commands and visualized the state of the load provided by the ToF sensors.
- Probe 20 completed the control logic of the prototype.
- Probe 23 introduced asyncio library to allow seemingly simultaneous actions in the Python code. Python version had to be changed to 3.6 to support the asyncio library.
- Probe 3 brought together and presented the final proof-of-concept prototype to company representatives for evaluation.

During the week, Probes 6, 21, 22, 23, and 20 were worked together to finalize the prototype. The final prototype, Probe 3, combined the results of Probes 6, 7, 9, 15, 16, 17, 19, 20, 21, 22, 23, 30, 31, 36, and 37.

## 5. Insights, Discussion, and Generated Hypotheses

The purpose of this study was to create a proof-of-concept prototype for automating a particularly hard and heavy industrial lift using low-cost sensors and to develop hypotheses as a basis for future work and lay the foundation for researching product development of connected physical devices through the Industrial Internet. The reason for framing insights in the form of hypotheses is that it operationalizes other researchers and practitioners to further explore, test, and build on them. Creating a complete proof-of-concept prototype from an idea to a presentable piece of work with industry partners is a very educative experience. The predisposition towards the project was fairly skeptical from the parties involved, but because of the low cost of involvement and possible high return, the case company opened their doors and presented their challenges thoroughly. During and after the project, the case company raised its interest towards the solution and the proof-of-concept prototype. This acts as a basis for our first hypothesis, that it is actually possible to prototype such large-scale products and operations/interactions connected to them. In future projects, creativity and scaling might be needed as our case has proven as well, for example a 30 ton stator was prototyped with a 60 kg block of metal with special machining (minimum load was required to be over 30 kg that the hoist would straighten itself under the load). Also, by offering developers access to similar equipment outside the manufacturing facilities, it becomes possible to try out new ideas without interference with production. This is also one of the core principles of Wayfaring in the concept phase.

Having reflected during and after the product development project we, the authors, generated five hypotheses based on the experiences and insights of the project, and the feedback of both the broader circle of researchers and the project companies. The first two are primarily interesting for the authors in this context and the other three are more general insights framed as hypotheses. Based on the time of seven working weeks and little economical input used for creating a functional proof-of-concept the first hypothesis is about prototyping:

**Hypothesis** **H1:** *Prototyping connected heavy industrial machinery is possible with low-cost and time involvement also in non-obvious environments.*

The second hypothesis addresses the challenge of decision making within the new product development of Industrial Internet. The applied field of industry is particularly challenging as it is new emerging field. Foreseeing which technologies to focus on and what would work is very difficult, that it is why it is proposed to try out the methodology in order to gain more information about the actual potential solutions in each context. Ersen et al. states the problem and a solution: “The decision makers need more informative models to evaluate the projects that have a high degree of uncertainty” [25]. While this might be true, we hypothesize that by acquiring more information about the decision we can reach the same goal. Thus, by prototyping the project with a pre-project, the investment decision may become easier by gaining more precise information about the risks, the costs, and the potential benefits. Showing the proof-of-concept prototype to the middle management of the company allowed us to present the next hypothesis about lowering the risk of the investment decision by creating a pre-project before investing.

**Hypothesis** **H2:** *Investment decision for heavy Industrial Internet products becomes easier with concrete data from a proof-of-concept prototype by creating knowledge about the investment risk and the value proposition.*

Although the crane produces a lot of data about its condition, it was unclear how to make the best use of this data. However, by building APIs for the existing digital twin, the crane company basically enabled the physical crane to act as a platform for the third party developers to build and sell specific applications for the use of the crane [26]. The authors were granted this access through their own refurbished crane and the development was carried out by the researchers together with the crane manufacturer in order to demonstrate both the technology and methodology capabilities. The authors acting as the third party for the companies and using their knowledge to create a specialized application for the needs and wishes of a client company of the crane company can be seen as an opportunity that anyone could use to found a company offering similar solutions for the broader audience of the crane users:

**Hypothesis** **H3:** *Platformization of the products will enable third parties’ offering of applications, product increases in value, when others make applications on top of it.*

During and after the project, it was clear how much easier and faster it is to prototype with APIs created by high-level programming languages (such as Python vs. C). Industry level communication standards such as OPC-UA are a bit cumbersome to use, but after creating a Python class for controlling the crane, it was self-evident that it was pleasant and fast to operate the crane through the abstraction (because the developer does not need competence in programming crane PLC). There seems to be a lot to learn from the modern web development practices to industrial product development and programming:

**Hypothesis** **H4:** *Using existing APIs enables fast prototyping, and bringing ‘developer culture’ from the ‘software world’ to the ‘physical world’ enables faster prototyping/product development cycles.*

While developing a control system for a potentially dangerous machine, we had to solve numerous challenges such as considering the unauthorized access to the crane or mitigating the effect of bugs in the programming.

**Hypothesis** **H5:** *Connecting heavy Industrial Internet products (such as a crane) with a potentially dangerous third party ’thing’ can be done safely by limiting control capacity in the API of the crane (Sandbox).*

### 5.1. Limitations

As mentioned before, participation both in the reflection and working is a very demanding research approach and one limitation from this approach is so-called ‘researcher bias’ [27]. For this reason, we provide only hypotheses that need to be validated further, instead of absolute conclusions. On one hand, when researchers act as team members, it is a given that the data received is not filtered or altered in any way before reaching the researchers. On the other hand, this sets high requirements for the individuals practicing this type of research. They have to have both personal and professional skills to develop the products while still researching objectively the raw material of the research: the project or the process of the respective case. This sets high standards for documenting the project with adequate detail since the flow of information is quite extensive. The same limitations are shared with participation action research [28].

### 5.2. Reflections on the Working Method

Having been forced to follow the structure of the work due the distance to the location of the crane, the authors ended up reflecting the pros and cons of the way of working, dividing the tasks into one-week intensive periods with roughly one month in between.

On the positive side, there is an argument that time is not wasted awaiting delivery or any external services for whatever needs to be purchased for the project, since there is a natural cycle of working and not working. It is also easier for the managers to have a fixed week on one task than to practice product development every now and then. The distance and time between intensive weeks enables the team to reflect upon the solutions with time, which could be otherwise more difficult if the project participants are involved constantly.

On the negative side of this way of working lies the fatigue caused by forgetting the last working period and ‘starting over’ every time a new period starts. The project also spreads over a longer period of time instead of only the absolute effective time that is used for the project. This is also a critique for the project; since the longer the projects take, the more unreliable conclusions and theories can be extracted from the data.

## 6. Outlook

After the project, the development platform has been utilized in one three-month master level student project that added new sensors to the platform. Furthermore, the platform has inspired the initiation of a new project aimed at eliminating load sway completely using an artificial neural network. Research-wise, these hypotheses need to be tested using proper research methodology by different fields that might benefit from this approach of thinking and decision making.

In the future, if all products have their simulation based digital twins with working data interfaces, virtual prototyping could have a more significant role in early product development. Functional properties of virtual prototyping can be compared to those of virtual commissioning of manufacturing systems, which has unfortunately been seen as time consuming [29]. However, in many product development cases the required digital twin based virtual prototype is much simpler than a full manufacturing system, which reduces the amount of labor. The digital twin-based prototyping might well be a convenient tool for new product development if manufacturers start providing simulation models with programming interfaces, as they have started providing 3D models of their products. However, the most important decision is to choose a suitable method for each probe that maximizes learning and minimizes the cost and time used for development in order to achieve better results at the early stage of new product development.

## Figures and Tables

**Table 1:** List of probes that were identified.

Basic Information for the Probes			Active Weeks							
**Number**	Probe description	Success	0	1	2	3	4	5	6	7
**Not discipline-specific probes**										
**1**	Benchmarking and brainstorming	-	x	-	-	-	-	-	-	-
**2**	Site visit at assembly company	-	-	x	-	-	-	-	-	-
**3**	Final proof-of-concept presentation	-	-	-	-	-	-	-	-	x
**User interface probes**										
**4**	Graphical UI (with HTML)	x	-	-	-	x	-	-	-	-
**5**	Stepwise UI (with command line, curses)	x	-	-	-	-	x	-	-	-
**6**	Phases/stepwise UI	v	-	-	-	-	-	-	x	x
**Software probes**										
**7**	Review: Language selection	v	-	x	-	-	-	-	-	-
**8**	Review: Method for controlling the crane	-	-	x	-	-	-	-	-	-
**9**	Decision: OPC UA is the API	v	-	x	-	-	-	-	-	-
**10**	Using OpenCV as image detection software	x	-	-	x	-	-	-	-	-
**11**	Review of OPC UA tools	-	-	-	x	x	x	-	-	-
**12**	Using Python as programming language	-	-	-	x	x	x	x	x	-
**13**	Review: Tools for UI visualization (on demand)	-	-	-	-	x	x	-	x	-
**14**	Using Flask as software tool for UI	-	-	-	-	x	-	-	x	-
**15**	Integrating ToF libraries to python code	v	-	-	-	x	-	-	-	-
**16**	Setting up the OPC UA API of the crane	v	-	-	-	x	x	x	-	-
**17**	Setting up FreeOpcUa library	v	-	-	-	-	x	-	-	-
**18**	Testing joystick software tool for UI	x	-	-	-	-	-	x	-	-
**19**	Developing "crane.py" class for python	v	-	-	-	-	-	x	x	-
**20**	Developing control software	v	-	-	-	-	-	x	x	x
**21**	Tornado as SW tool for UI	v	-	-	-	-	-	x	x	-
**22**	Setting up WebSocket	v	-	-	-	-	-	-	x	x
**23**	Introducing Python 3.6 and asyncio library	v	-	-	-	-	-	-	-	x
**Electronics probes**										
**24**	Testing light to help measuring	x	-	x	-	-	-	-	-	-
**25**	Review: sensors and measuring concept	-	-	x	x	x	-	-	-	-
**26**	Review: self-calibration method	x	-	x	-	-	-	-	-	-
**27**	Review: computation hardware	-	-	x	-	-	-	-	-	-
**28**	Using lapmiddle as computing platform	-	-	-	x	x	x	x	-	-
**29**	Using camera as measurement sensor	x	-	-	x	-	-	-	-	-
**30**	Setting up ToF sensors	v	-	-	-	x	-	x	-	-
**31**	Using Raspberry Pi as computing platform	v	-	-	-	-	x	x	-	-
**32**	Using Arduino as computing platform	x	-	-	-	-	-	x	-	-
**Mechanics probes**										
**33**	Bottles and cans as test rig	x	-	x	x	-	-	-	-	-
**34**	Oil barrel as test rig	x	-	-	x	-	-	-	-	-
**35**	Planning the machined test rig	v	-	-	-	x	-	-	-	-
**36**	Assembling the machined test rig	v	-	-	-	-	x	x	-	-
**37**	Setting up sensor platform	v	-	-	-	-	-	x	-	-

**Table 2:** Review of possible sensors as result of Probe 25.

Sensor Type	Model	Usability	Price	Specifications	Link
Laser (triangulation)	Keyence IL-300 (example)	Analog output, requires ADC converter to connect to Rapberry Pi.	Several hundred to thousands USD	Repeatability: 30 μm, Linearity: ±0.25 of F.S. (Keyence IL-300)	https://www.keyence.com/products/measure/laser-1d/il/models/il-300/index.jsp
Infra-red	Sharp GP2Y0A21YK (example)	JST connector, analog output	$13.95	3.1 V at 10 cm to 0.4 V at 80 cm	https://www.sparkfun.com/products/242
Ultrasound	HRXL-MaxSonar-WR (example)	Arduino and Raspberry Pi compatible. Sensor outputs: Analog Voltage, Serial, Pulse Width.	$99.95	Resolution of 1mm	https://www.sparkfun.com/products/11724
Camera with feature detection	Logitech C920 webcam	USB connection to Raspberry Pi. Requires feature detection, for example using OpenCV library.	$79.99 (already available at laboratory)	Dependent on distance, pixel count and camera vision software.	https://www.logitech.com/en-us/product/hd-pro-webcam-c920
Eddy-current	Metrix MX2030 (example)	Analog output, requires ADC converter to connect to Rapberry Pi.	One to several hundred USD	Typical range 2 mm. Up to 40 mm available.	http://www.metrixvibration.com/products/proximity/digital-proximity-system/product/657/mx2030-probe-series
Thermal camera	FLiR Dev Kit	SPI port, works with Arduino or Raspberry Pi	$229.95	resolution of 80 × 60 pixels	https://www.sparkfun.com/products/13233
UWB radar	X4M200	Python API	$249.00	detection zone of 0.4–5.0 m	https://shop.xethru.com/x4m200
Laser (Time of Flight)	VL53L0X board from Polulu	I2C interface (available in Raspberry Pi). Open source library available for Python.	$9.95	Resolution 1 mm. Accuracy ±3 to ±10% depending on conditions.	https://www.pololu.com/product/2490
